# Visual Field Testing with Head-Mounted Perimeter ‘imo’

**DOI:** 10.1371/journal.pone.0161974

**Published:** 2016-08-26

**Authors:** Chota Matsumoto, Sayaka Yamao, Hiroki Nomoto, Sonoko Takada, Sachiko Okuyama, Shinji Kimura, Kenzo Yamanaka, Makoto Aihara, Yoshikazu Shimomura

**Affiliations:** 1 Department of Ophthalmology, Kindai University, Faculty of Medicine Osaka-Sayama City, Osaka, Japan; 2 CREWT Medical Systems, Inc., Tokyo, Japan; 3 Department of Ophthalmology, Graduate School of Medicine and Faculty of Medicine, The University of Tokyo, Tokyo, Japan; The University of Melbourne, AUSTRALIA

## Abstract

**Purpose:**

We developed a new portable head-mounted perimeter, “imo”, which performs visual field (VF) testing under flexible conditions without a dark room. Besides the monocular eye test, imo can present a test target randomly to either eye without occlusion (a binocular random single eye test). The performance of imo was evaluated.

**Methods:**

Using full HD transmissive LCD and high intensity LED backlights, imo can display a test target under the same test conditions as the Humphrey Field Analyzer (HFA). The monocular and binocular random single eye tests by imo and the HFA test were performed on 40 eyes of 20 subjects with glaucoma. VF sensitivity results by the monocular and binocular random single eye tests were compared, and these test results were further compared to those by the HFA. The subjects were asked whether they noticed which eye was being tested during the test.

**Results:**

The mean sensitivity (MS) obtained with the HFA highly correlated with the MS by the imo monocular test (R: r = 0.96, L: r = 0.94, *P* < 0.001) and the binocular random single eye test (R: r = 0.97, L: r = 0.98, *P* < 0.001). The MS values by the monocular and binocular random single eye tests also highly correlated (R: r = 0.96, L: r = 0.95, *P* < 0.001). No subject could detect which eye was being tested during the examination.

**Conclusions:**

The perimeter imo can obtain VF sensitivity highly compatible to that by the standard automated perimeter. The binocular random single eye test provides a non-occlusion test condition without the examinee being aware of the tested eye.

## Introduction

Visual field (VF) testing is essential in diagnosing and monitoring many ophthalmological and neurological diseases. Automated perimeters such as the Humphrey Field analyzer (HFA) (Carl Zeiss Meditec, Dublin, CA) and Octopus perimeter (Haag-Streit, Koeniz, Switzerland) have been widely used in the field of standard automated perimetry (SAP). However, most of these perimeters are stationary type devices that need to be used in a dim testing room with light control. In addition to issues such as portability and space restriction for the standard automated perimeters, patients with special physical conditions may experience difficulty or discomfort trying to physically adapt themselves to the stationary type devices during the test. Therefore, a patient-oriented perimeter with better flexibility in performing the test is a pressing need and a head-mounted perimeter appears to be ideal.

Several head-mounted perimeters that do not require a dark room for VF testing have been previously developed for laboratory-based studies[1–3]. However, there is no commercially available head-mounted perimeter devised for clinical setting. We recently developed a new portable head-mounted perimeter named “imo” (CREWT Medical Systems, Tokyo, Japan). imo can perform VF testing without a dark room and under test conditions compatible with those for SAP. It also allows patients to be tested with better comfort at any location. Moreover, as a unique feature of this device, the test target is randomly presented to either eye without occlusion and without the examinee being aware of which eye is being tested (the binocular random single eye test).

In this study, we reported the detailed features of our new device. As a pilot study, VF sensitivity values obtained using the imo monocular and binocular random single eye tests were compared with the HFA results in patients with glaucoma.

## Subjects and Methods

### The head-mounted perimeter ‘imo’

**Fig 1:**
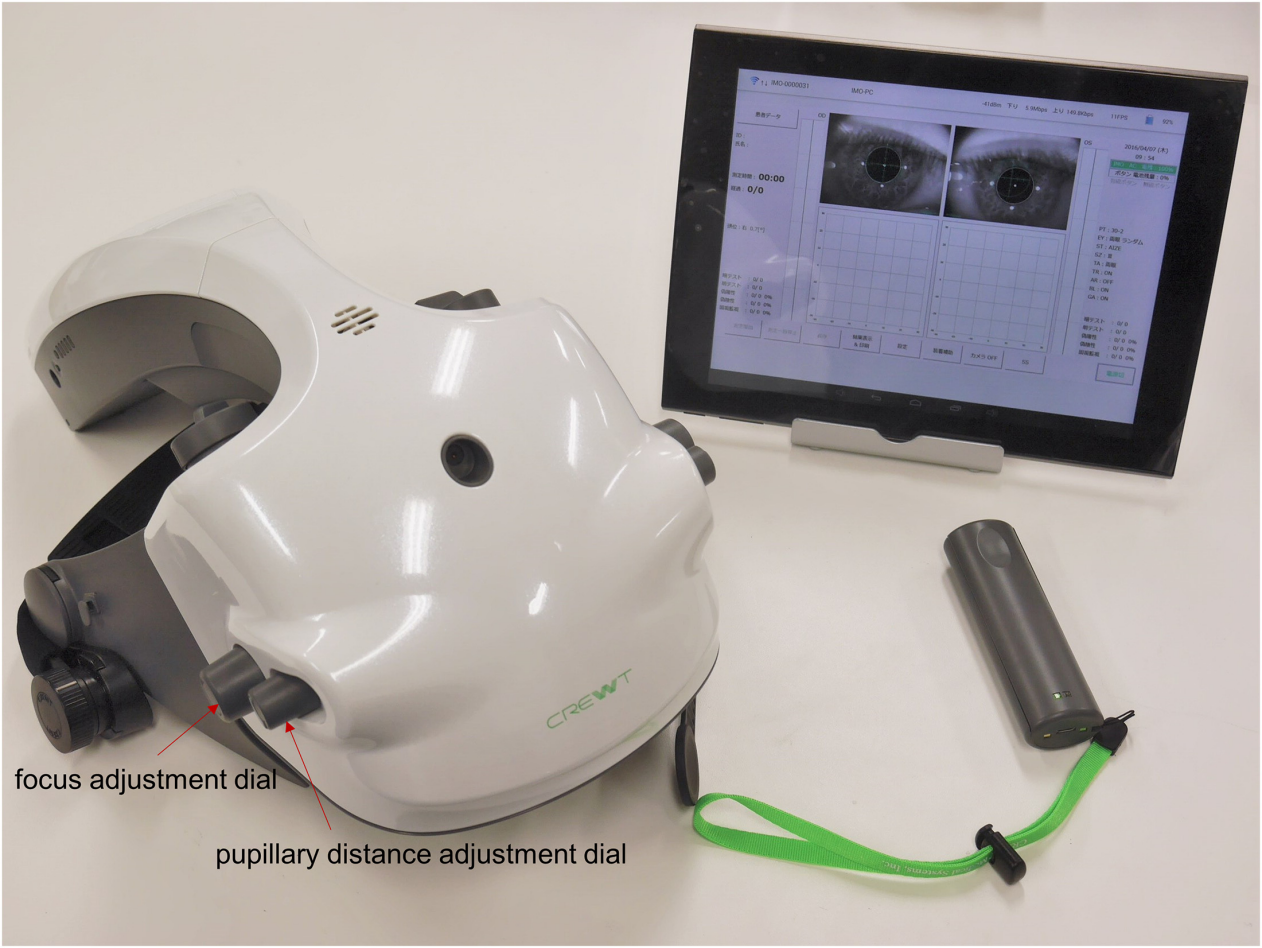
The head-mounted perimeter imo. The perimeter imo consists of a main perimeter unit, a user control tablet, and a patient response button.

The head-mounted perimeter imo consists of a main perimeter unit, a user control tablet, and a patient response button. A computer unit and a lithium-ion battery are built in the perimeter unit (W22 cm × D38 cm × H24 cm, 1.8 kg). In a VF test, the examiner operates the control tablet connected to the perimeter unit by Wi-Fi and patient’s responses are obtained using the response button connected by Bluetooth (Fig 1). With these integrated functions, imo realizes a portable high-performance perimeter as compared with the conventional devices. A stationary stand for imo is prepared during the test if the examinee chooses not to wear the device for any special physical reason.

**Fig 2:**
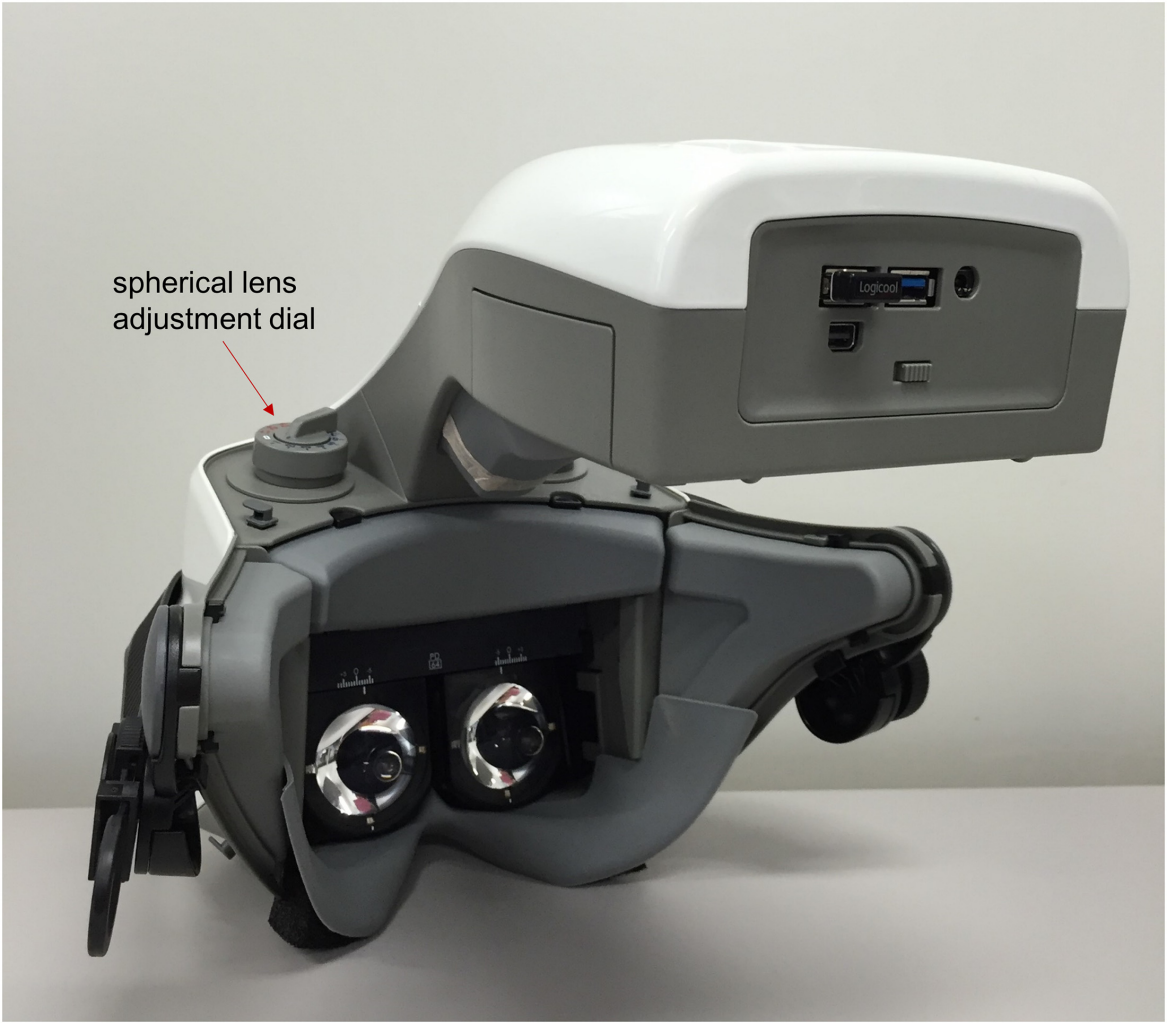
The perimeter unit. The perimeter imo has completely isolated optical systems for the right and left eyes. Stimulus presentation is also independently performed for each eye.

The right and left optical systems in the perimeter unit are completely separated and stimulus presentation and pupil monitoring are independently performed for each eye (Fig 2). The optical system is a wide-angle lens system which can measure the VF within 35°from the fovea. With the function of distortion and field curvature corrections, stimuli can be accurately generated and presented. A telecentric optical system is introduced to equalize the central and peripheral light intensities.

**Fig 3:**
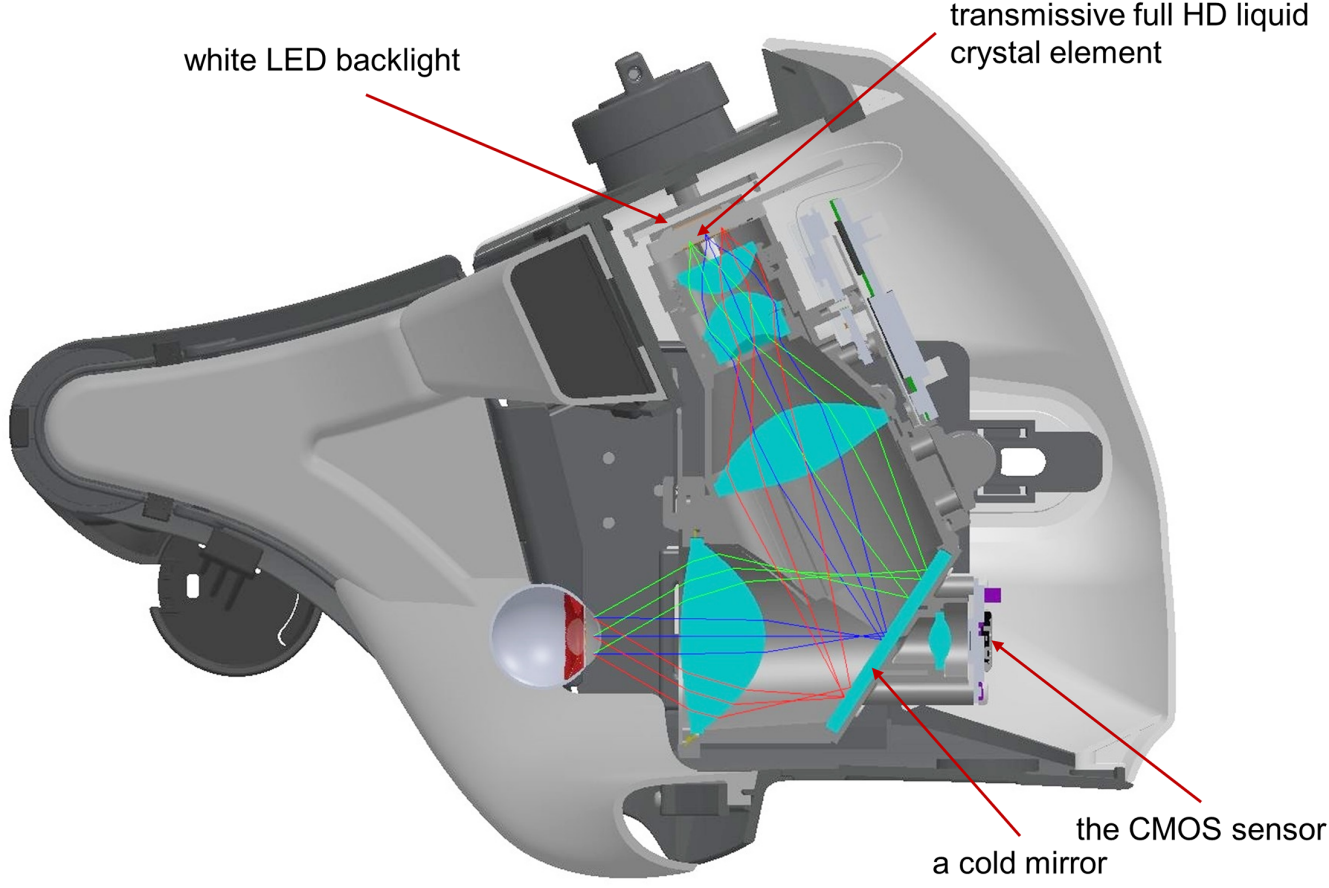
A cross-section image showing the structure of the perimeter unit. A test target is displayed on the full HD transmissive liquid crystal displays with high intensity LED backlights for the two eyes. Both pupils are illuminated by near infrared LEDs and these images are monitored by the SXVGA resolution CMOS image sensor.

During the test, a test target was displayed using two sets of full high-definition (HD) transmissive liquid crystal displays and high intensity light emitting diode (LED) backlights separately for the right and left eyes. A test target luminance of 0.032–3183 cd/m^2^ (0.1–10000 asb) with a background luminance of 10 cd/m^2^ (31.4 asb) was generated using 10 bit resolutions, and the stimulus duration was 200 msec. The temporal resolution of the transmissive LCD display was 60 Hz and the stimulus intensity reached to a constant luminance level within one frame (1.67 msec). Test targets used the Goldmann size I to V, or any other optimal sizes and shapes available. The spatial resolution of the full HD transmissive LCD used in this device was 1920 x 1080 for each eye. Because the VF beyond the central 35°was masked on the LCD screen, 79800 pixels were actually used for testing. A Goldmann target size III (0.431° visual angle) was displayed using 37 pixels and used as the standard target size in this study. Due to the limitation of the display resolution, target size II and I were respectively displayed using 12 and 2 pixels and it is not currently possible to present a circular size I stimulus. Both right and left pupils were illuminated by near infrared LEDs and these images were obtained using the SXVGA resolution (1280 X 960 pixel) complementary metal–oxide–semiconductor (CMOS) sensor with a maximum frame rate of 60 Hz (Fig 3).

During the examination, both pupil images were continuously monitored at a frame rate of 30 Hz and the images could be used for an eye tracking system. In this study, the subjects were not tested with the eye-tracking mode. Using the spherical lens adjustment dials shown in Fig 2, the lens position inside the perimeter was mechanically moved and a spherical lens correction within the range of -9 to +3 diopters could be performed without using any additional trial lenses. Furthermore, astigmatic correction could be achieved as well using an additional removable magnetic cylindrical lens system. The actual distance between the center of the cornea surface and the lens was 17.5 mm and patient’s viewing distance was set at 1 meter. Using the focus adjustment dial shown in Fig 1, this distance was adjustable within a control range of ± 3 mm according to the shape of the examinee’s face. Even if this distance was adjusted to ± 3 mm, with the imo optical system, the visual angle of the target size would only vary within ± 0.4%.

**Fig 4:**
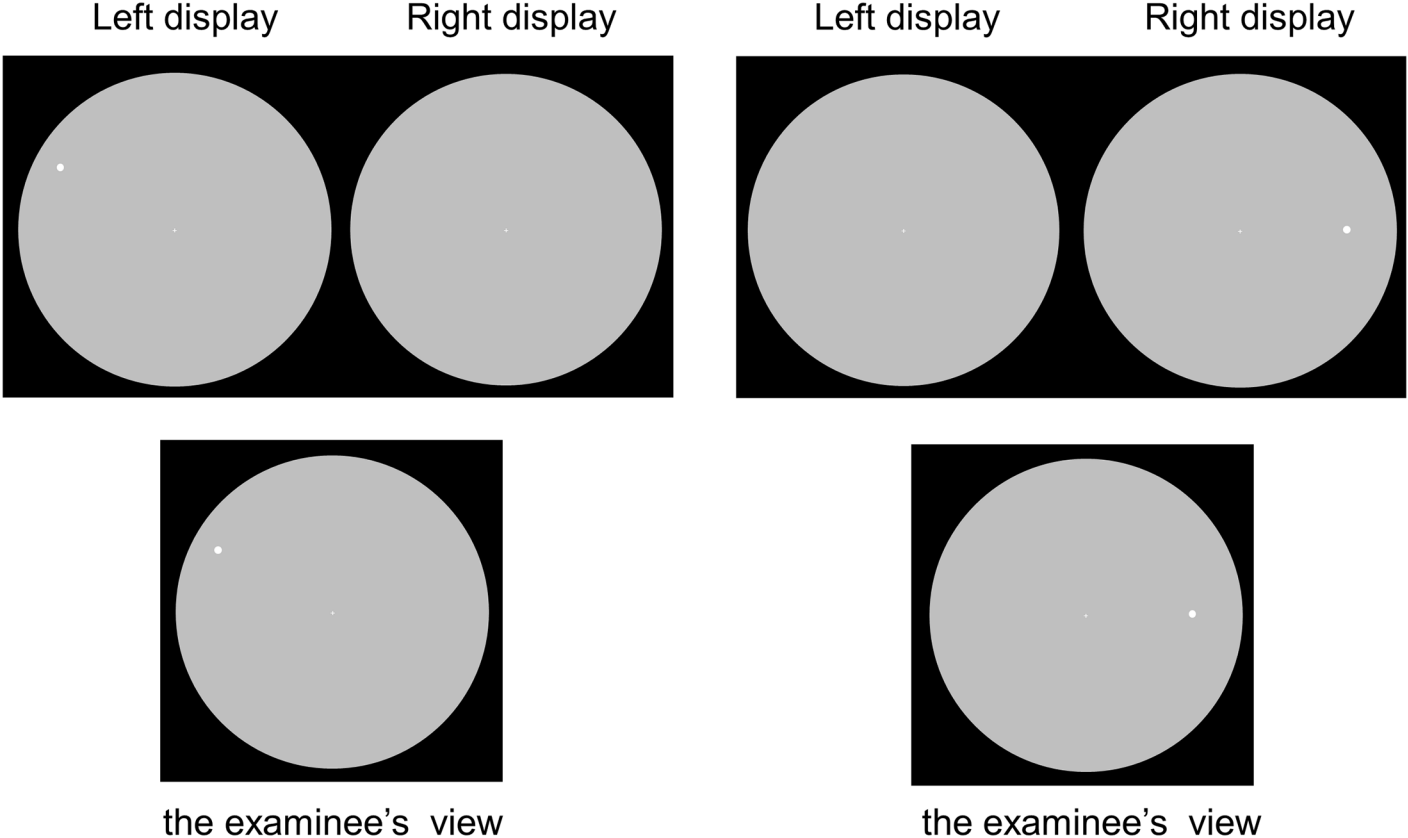
Target presentation and the examinee’s view during the binocular random single eye test. The test target was presented randomly to either eye under a non-occlusion condition and the patient was not aware of which eye was being tested. To better demonstrate this test with visible targets, target size V is used in this figure although target size III was the actual size used in this study.

This study used a 30–2 test pattern with 4–2 dB bracketing strategy. The test patterns used for imo are compatible with those for the HFA 30–2, 24–2, 10–2 and 24+ test programs with additional test points in the central 10°VF of the 24–2 program. For thresholding algorisms, the conventional 4–2 dB bracketing strategy and a modified Zipper Estimated Sequential Testing (ZEST) named AIZE (Ambient Interactive ZEST) are available. Furthermore, a binocular random single eye test that is a new testing approach is created for imo as the special feature. Conventional perimetry usually tests the right and left eyes separately. imo not only can test the right and left eyes separately but also presents the test target randomly to either eye under a non-occlusion condition without the examinee being aware of which eye is being tested (Fig 4).

### Subjects

Subjects were 40 eyes of 20 patients with glaucoma (mean age, 60.2 ± 5.8 years; SE, -2.93 ± 2.58 D). The 40 eyes included 16 eyes with primary open angle glaucoma (POAG), 14 eyes with normal tension glaucoma (NTG), 3 eyes with primary angle closure glaucoma (PACG), 3 eyes with secondary glaucoma, and 4 normal contralateral eyes of the patients. Sixteen eyes were in the early stage of glaucoma (MD ≤ 6 dB), 10 eyes were in the moderate stage (6 dB< MD ≤ 12 dB), and 10 eyes were in the advanced stage (12 dB > MD). The exclusion criteria were: best corrected visual acuity < 1.0, refractive error < 6 D sphere and < 2.50 D cylinder, pupil diameter of < 3.0 mm, an ocular surgical history, ocular diseases other than glaucoma that might cause VF loss, and systemic diseases which were likely to affect the patient’s visual functions. Patients with the HFA results with more than 15% positive or negative catch trials and fixation loss, and patients with fusion dysfunction due to strabismus were also excluded. The diagnostic criteria for glaucomatous VF abnormality were as follows: the pattern deviation probability plot that showed a cluster of 3 or more nonedge-contiguous points having sensitivity with a probability of < 5% in the upper or lower hemifield with at least 1 point with a probability of < 1%. The diagnosis of glaucoma was based on the presence of typical glaucomatous optic disc changes, clear nerve fiber bundle defects, and corresponding glaucomatous VF abnormalities by the HFA.

All the subjects underwent the HFA SITA standard 30–2 test, and the 30–2 monocular and binocular random single eye tests by imo. The order of the imo monocular and binocular random single eye tests was randomized. VF testing using imo was performed in a regular clinical office setting with an adaptation time of at least 5 minutes including the time for device setting. The examination would be interrupted when the test duration reached to 10 minutes. The results of the imo monocular and binocular random single eye tests were compared with the HFA results. Comparison of the mean sensitivity (MS) values obtained by both methods was performed using the Tukey test and Spearman's rank correlation coefficient. The regression lines were calculated using a Deming regression method. Bland-Altman plots [4] were used to assess the agreement between the results of the imo monocular and binocular random single eye tests. The subjects were asked if they noticed which eye was being tested during the measurement.

This study followed the tenets of the Declaration of Helsinki, and all the participants provided written informed consent after the ethics committee of Kindai University Faculty of Medicine (no. 26–239) had approved the study.

## Results

### Comparison of test results

**Fig 5:**
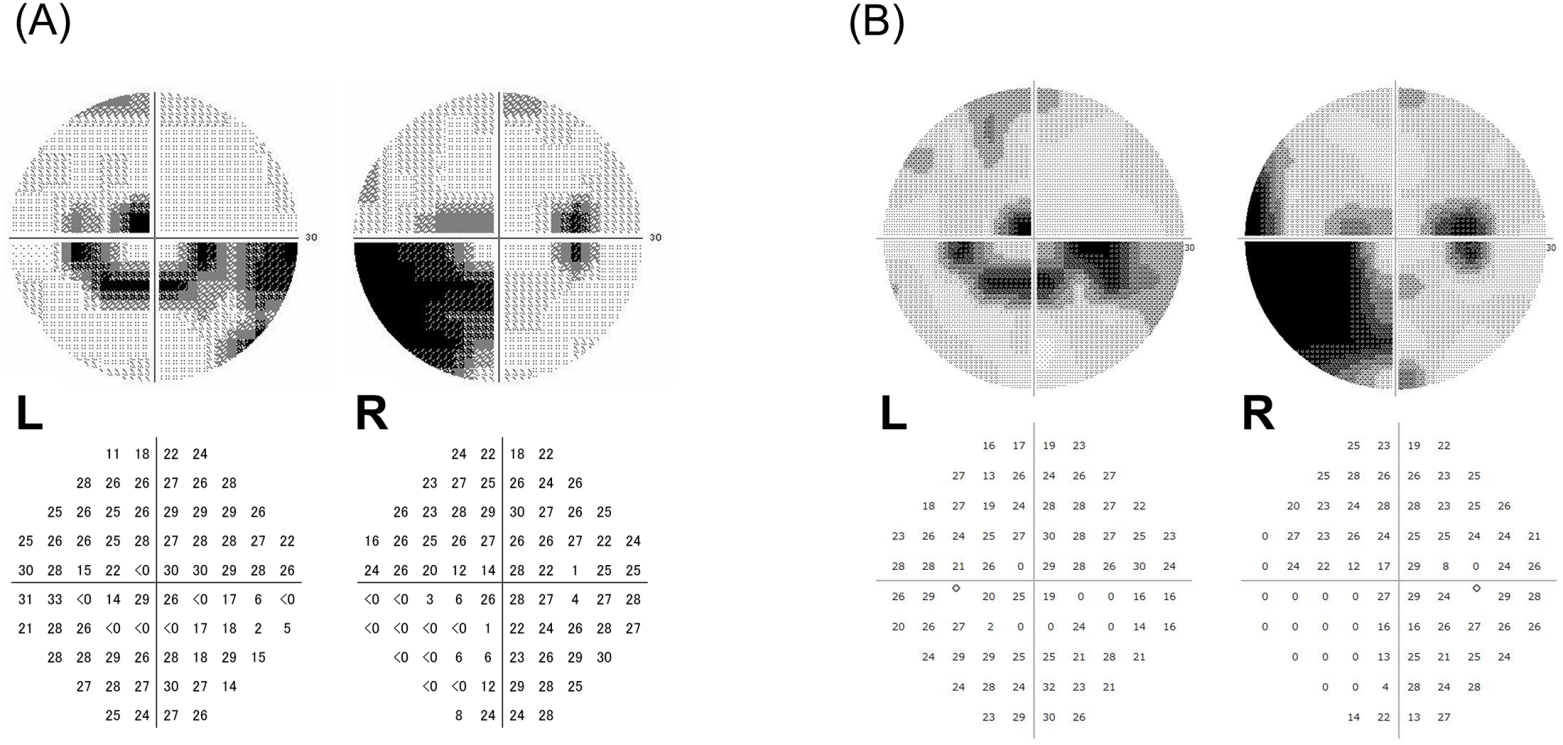
Description of the glaucomatous VF defects by grey scale and actual values for a 61-year-old male with POAG. **(A)** A deep lower nasal sensitivity loss in the right eye, a lower arcade scotoma and an upper small scotoma near the fixation point in the left eye were detected by the HFA. **(B)** Similar defects were detected using the imo binocular random single eye test.

Fig 5 shows a case of a 61-year-old male with POAG in both eyes. A deep lower nasal sensitivity loss in the right eye, a lower arcade scotoma, and an upper small scotoma near the fixation point in the left eye were detected by the HFA (Fig 5A). The imo binocular random single eye test also detected similar abnormalities in both eyes (Fig 5B).

### Comparison of the MS values

**Fig 6:**
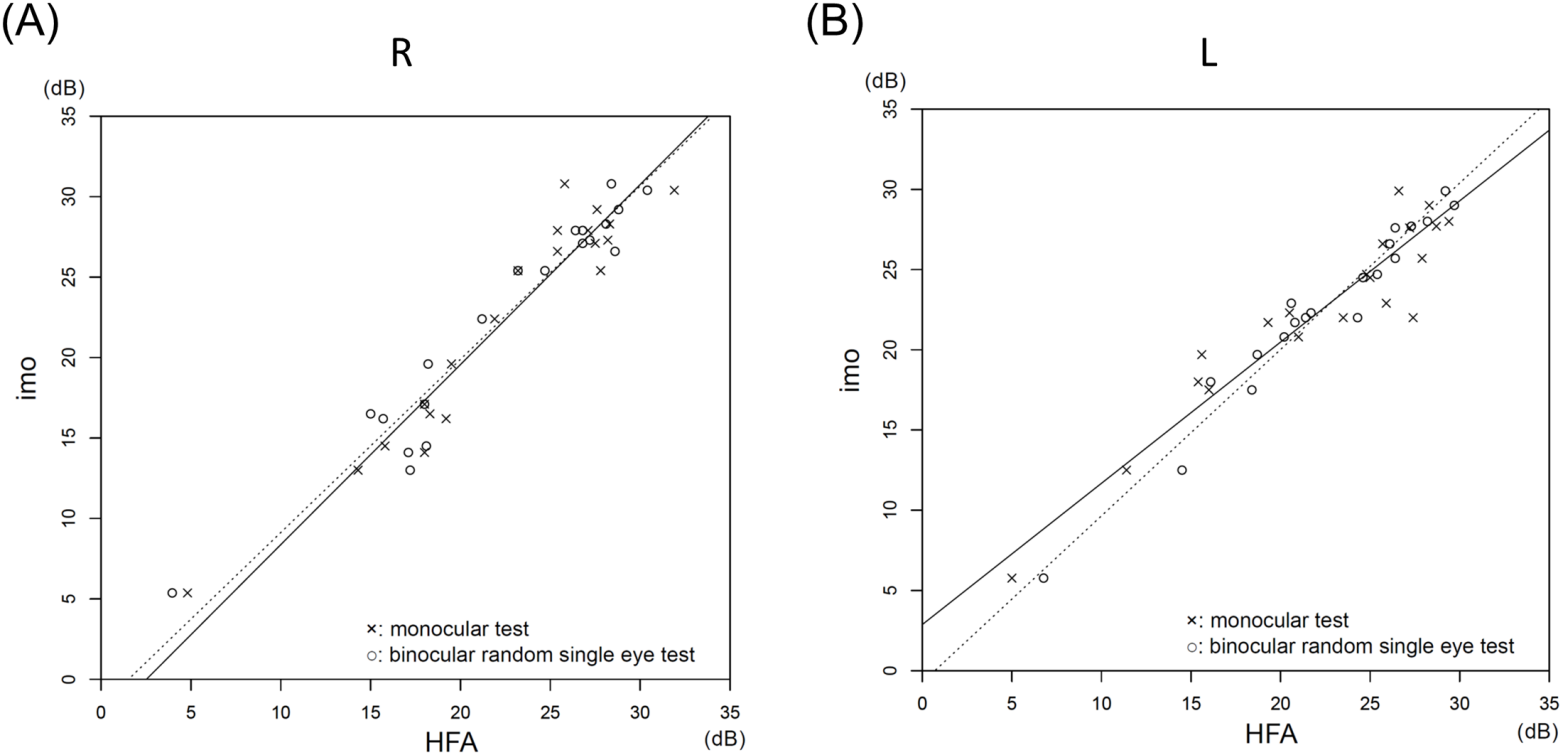
Correlations between the MS values by the HFA and the imo tests. **(A)** The MS values for the right eye by the HFA highly correlated with the values by the imo monocular test (the solid regression line: r = 0.96, *P* < 0.001) and the binocular random single eye test (the dotted regression line: r = 0.97, *P* < 0.001). The slopes of the Deming regression lines were 1.12 (95% confidence interval [CI], 1.01 to 1.37) for the solid line and 1.08 (95% CI, 0.97 to 1.32) for the dotted line. The intercepts were -2.82 (95% CI, -8.48 to -0.37) for the solid line and -1.64 (95% CI, -7.67 to 1.12) for the dotted line. **(B)** Similarly, the MS values for the left eye by the HFA highly correlated with the values by the imo monocular test (the solid regression line: r = 0.94, *P* < 0.001) and the binocular random single eye test (the dotted regression line: r = 0.98, *P* < 0.001). The slopes of the Deming regression lines were 0.88 (95% CI, 0.70 to 0.98) for the solid line and 1.37 (95% CI, 0.87 to 1.11) for the dotted line. The intercepts were -2.88 (95% CI, 0.92 to 7.24) for the solid line and -0.71 (95% CI, -2.65 to 3.34) for the dotted line.

**Fig 7:**
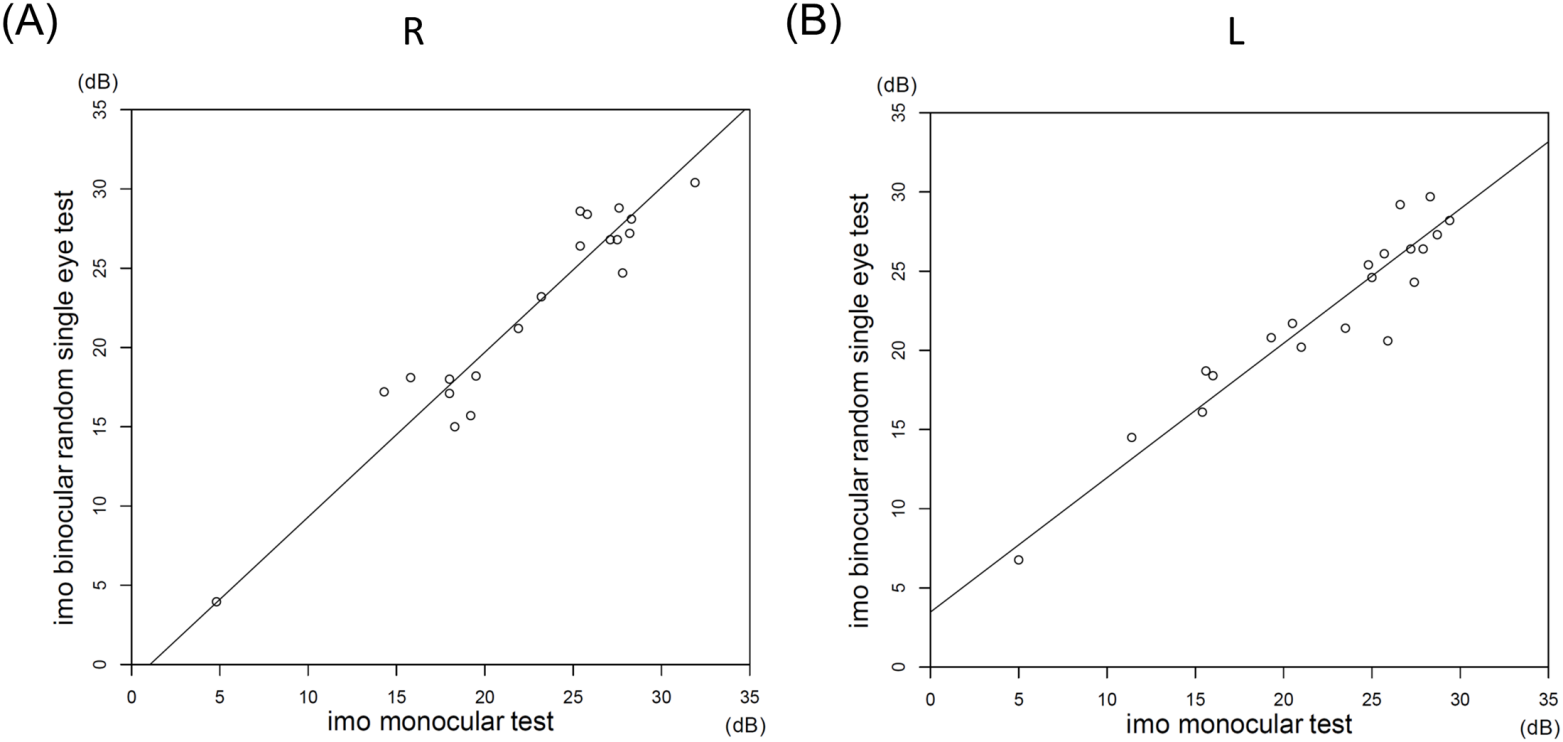
Correlations between the MS values for the right and left eyes by the two imo tests. The MS values by the two imo tests highly correlated for the two eyes (R: r = 0.96, L: r = 0.95, *P* < 0.001). The slopes of the Deming regression lines were 1.04 (95% CI, 0.89 to 1.26) for the right eye and 0.85 (95% CI, 0.71 to 0.95) for the left eye. The intercepts were -1.09 (95% CI, -6.30 to 2.66) for the right eye and 3.47 (95% CI, 0.92 to 6.35) for the left eye.

The MS values for the right eye and the left eye obtained by the HFA, the imo monocular test, and the binocular random single eye test did not significantly differ (*P* = 0.99, the Tukey test; Table 1). The MS values by the HFA highly correlated with the MS values by the imo monocular test (R: r = 0.96, L: r = 0.94, *P* < 0.001; Spearman rank-order correlation) and with the values by the binocular random single eye test (R: r = 0.97, L: r = 0.98, *P* < 0.001; Spearman rank-order correlation) (Fig 6). The MS values by the imo monocular and binocular random single eye tests also highly correlated (R: r = 0.96, L: r = 0.95, *P* < 0.001; Spearman rank-order correlation) (Fig 7).

**Table 1:** The MS values obtained by the HFA and imo tests.

	HFA	monocular test (imo)	binocular random single eye test (imo)	*p-value*
the right eye	22.3 ± 7.2	22.4 ± 6.4	22.2 ± 6.7	0.99
the left eye	22.4 ± 5.9	22.2 ± 6.6	22.3 ± 5.7	0.99

**Fig 8:**
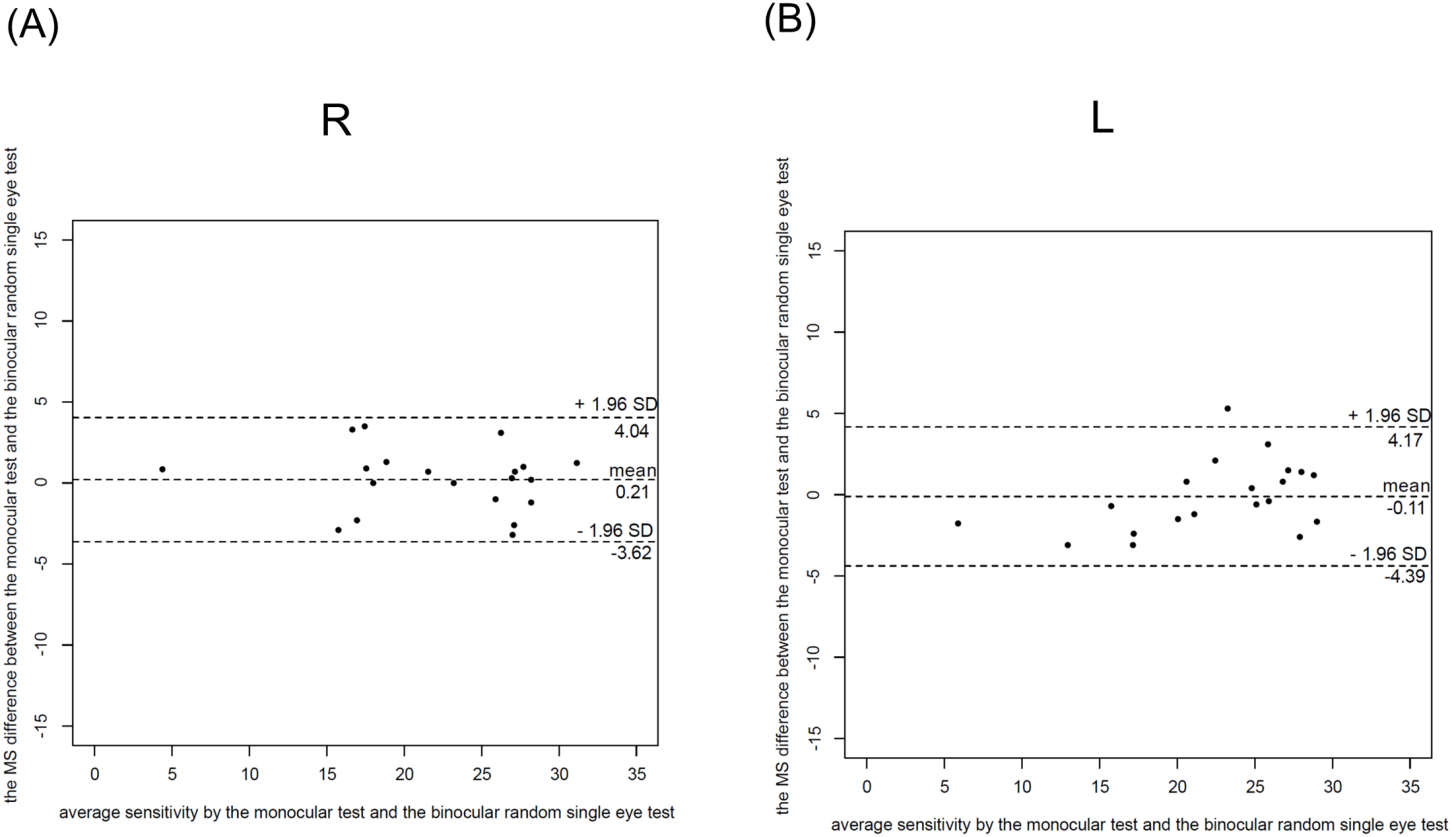
Bland-Altman plots of the MS differences for the right and left eyes between the two imo tests. Bland-Altman plots revealed MS differences of 0.21 dB (95% limits of agreement between -3.6 dB and 4.04 dB) for the right eye and -0.11 dB (95% limits of agreement between -4.39 dB and 4.17dB) for the left eye between the two imo tests.

Bland-Altman plots revealed MS differences of 0.21 dB (95% limits of agreement between -3.6 dB and 4.04 dB) for the right eye and -0.11 dB (95% limits of agreement between -4.39 dB and 4.17dB) for the left eye between the imo monocular and binocular random single eye tests (Fig 8).

The average test durations were 17.30 ± 1.25 min for the imo binocular random single eye test and 16.0 ± 1.21 min for the HFA test for the right and left eyes. In addition, no subject could detect which eye was being tested during the imo examination.

## Discussion

Our study clearly showed that the new head-mounted perimeter imo can perform VF testing without a dark room and detect glaucomatous VF abnormalities compatible to the HFA results. Moreover, the equipped binocular random single eye test requires no occlusion for testing and produces results highly correlated with the results by the conventional monocular method. The head-mounted perimeter imo appeared to be a promising new perimetric method.

The new perimeter imo has a great advantage: VF testing can be performed under a non-occlusion condition. The conventional monocular test usually requires the untested eye to be occluded. Possible problems caused by the test condition of occlusion have been previously reported. It is suspected that when the dominant eye is occluded, an inhibitory response in the non-occluded eye such as the Ganzfeld blankout or rivalry can be triggered by a binocular interaction [5, 6] although the effect of such phenomena on the SAP result is minimum [6]. In frequency-doubling technology (FDT) perimetry, it is well known that the second eye tested has reduced sensitivity [7–10] and that the delayed light adaptation is the suspected cause for the second eye problem. In addition to these problems, patients sometimes experience visual disturbances in the tested eye with the fellow eye being occluded with an opaque patch during static perimetric testing. With the imo binocular random single eye test, patients are tested under a more comfortable condition with both eyes open and the above-mentioned problems caused by occlusion can be solved. Because the test target is randomly presented to either eye, patients will not be aware of which eye is being tested during the test. Therefore, the binocular random single eye test can also be used for detecting feigned blindness. Indeed, all the subjects in this study could not detect which eye was being tested during the examination. As verified by the Bland-Altman plots in Fig 8, the MS difference between the imo monocular test and binocular random single eye tests was very small. The alternation of the two tests was clinically acceptable because they were within the intersession variability in patients with glaucoma [11, 12].

However, there are some concerns regarding the binocular random single eye test such as the test duration. Theoretically, the test duration for the binocular random single eye test would be twice as long as the test duration for the conventional monocular test. In addition, to reveal the character of the binocular random single eye test results, we used the traditional 4–2 dB bracketing strategy in this pilot study to exclude any possible effect from the strategy used. Therefore, the examination would take a recess when the test duration reached to 10 minutes and this further prolonged the test duration. To solve this problem, imo has a modified ZEST algorism named AIZE that reduces the test duration by about 70% as compared with the duration using the 4–2 dB bracketing strategy.

Another issue of the binocular random single eye test is patient’s eye position. In this study, we excluded patients with a fusion problem caused by strabismus. If a patient cannot achieve fusion on the center right and left fixation targets for each eye before the examination, it will be difficult to perform the binocular random single eye test accurately and the testing shall be switched to the traditional monocular test. Although the imo monocular test cannot test both eyes randomly, it still has the advantage of a non-occlusion test condition. Because the right and left optical systems in imo are completely separated, the same background intensity for the tested eye is also available for the fellow eye without occlusion in an imo monocular test.

The perimeter imo is an easy-to-use device for VF testing and all the subjects in this study could easily wear this device. However, it may not be suitable for everyone. For example, patients with a neck injury will not be recommended to use this device. As an alternative solution, a stationary stand for imo is prepared during the test for those who choose not to wear the device for testing. With this stand, patients can be tested without wearing the device.

In recent years, population ageing has become a serious healthcare problem in Japan. With the feature of portability, imo has the potential to be the ideal device for patients who are receiving medical care at home. Even in hospital, imo can also be useful for patients who cannot undergo the regular perimetric testing due to their physical conditions. With imo, perimetric testing can be performed at the bed side and obtain test results compatible to those by the traditional SAP. Since imo does not require a dark room for testing, mass screening also becomes possible with this device.

This pilot study has several limitations. To understand the characteristics of this new device, a reliability comparison between the new and conventional devices will be necessary and informative. Besides, repeatability performance that requires further investigation is another important issue in perimetric methods. However, these could not be achieved in this study because of the limitation of the total test durations and the small number of subjects. As a start, we used VF sensitivity as a measure to evaluate the basic characteristics of imo and the method behind although the systematic bias in MS between the two instruments may not be clinically significant. In the future, we are planning additional normative and repeatability studies using the modified zest algorism with shorter test durations.

In conclusion, we have demonstrated that the newly developed head-mounted automated perimeter imo is an ideal device that performs perimetric testing regardless of the location and patient’s physical condition. More importantly, it obtains VF results highly compatible to those by the HFA in patients with glaucoma. With the equipped binocular random single eye test in imo, occlusion is no longer required. All these unique features of imo indicated that imo has the potential to be the ideal automated perimeter of the future.
